# Incidence of Gingival Black Triangles following Treatment with Fixed Orthodontic Appliance: A Systematic Review

**DOI:** 10.3390/healthcare10081373

**Published:** 2022-07-24

**Authors:** Zhwan Jamal Rashid, Sarhang Sarwat Gul, Muhammad Saad Shaikh, Ali Abbas Abdulkareem, Muhammad Sohail Zafar

**Affiliations:** 1 College of Dentistry, University of Sulaimani, Sulaymaniyah 46001, Iraq; zhwan.rasheed@univsul.edu.iq; 2 Department of Oral Biology, Sindh Institute of Oral Health Sciences, Jinnah Sindh Medical University, Karachi 75510, Pakistan; drsaadtanvir@gmail.com; 3 College of Dentistry, University of Baghdad, Baghdad 10011, Iraq; ali.abbas@codental.uobaghdad.edu.iq; 4 Department of Restorative Dentistry, College of Dentistry, Taibah University, Al Madina, Al Munawwarra 41311, Saudi Arabia; mzafar@taibahu.edu.sa; 5 Department of Dental Materials, Islamic International Dental College, Riphah International University, Islamabad 44000, Pakistan

**Keywords:** orthodontic treatment, gingival black triangle, alveolar bone loss, risk factor

## Abstract

This systematic review aimed to investigate the relation between orthodontic treatment (OT) and the incidence of the gingival black triangle (GBT) after completing treatment with a fixed orthodontic appliance, as well as the associated risk factors and the level of alveolar bone. Electronic and hand searches were conducted in three electronic databases for relevant articles published up to March 2022. Retrieved articles went through a two-step screening procedure, and the risk of bias (RoB) was assessed by the Joanna Briggs Institute checklists. The incidence of GBT after OT was set as the primary outcome, while the secondary outcomes were the risk factors associated with GBT and alveolar bone loss following OT. Out of 421 papers, 5 were selected for the final analysis. The RoBs of three studies were moderate and the remaining two were low. The incidence of GBT following OT ranged from 38% to 58%. In addition, three studies reported that alveolar bone loss was reduced significantly following OT and associated with GBT, while one study found the opposite. Regarding the risk factors associated with GBT, the reported results attributed GBT to several factors including age, tooth-related factors, treatment duration, and soft tissue factors. The analysis indicates an increased incidence of GBT following OT; however, a firm conclusion cannot be drawn. Additionally, it was not possible to reach a consensus on risk factors associated with GBT due to the heterogeneity of the data. Therefore, further randomized clinical trials are highly recommended to draw a firm conclusion.

## 1. Introduction

The classical reasons for seeking orthodontic treatment (OT) have changed over the last decades. Recently, individuals have sought OT to solve functional occlusal discrepancies as well as esthetic needs [1]. Until the early 1980s, patients were seeking OT mainly to correct malocclusion or restore normal occlusal function [2]; however, economic growth combined with changes in social norms have led to an increased focus on dental esthetic among adolescents and adults [3]. Considering patients’ esthetic needs, OT planning is more challenging for both orthodontists and periodontists; therefore, an interdisciplinary treatment plan is essential for managing associated periodontal issues before, during, and after OT [1].

In healthy subjects, OT is mostly associated with transient inflammation and a minute insult to the periodontium [4,5]. Furthermore, a healthy periodontium can withstand tooth movements during OT without the deterioration of periodontal tissues [6]. On the contrary, OT may also have unwanted effects on periodontal tissues such as root resorption [7], bone dehiscence [8], loss of soft tissue architecture, gingival recession, and the formation of gingival black triangles (GBT), which may dramatically jeopardize the esthetic outcome [1]. GBT (also called “open gingival embrasure”) is formed due to the loss of interdental papilla [9]. In addition, GBT may cause periodontal problems due to food retention and potential difficulty in mechanical plaque control [10]. Therefore, the prevention of GBT formation by preserving the interdental papilla, especially in the esthetic zone, must be considered during OT [11]. Furthermore, understanding the etiopathophysiology of GBT and developing an appropriate treatment plan is crucial to decreasing the incidence and severity of GBT following OT [10].

The causes of GBT are multifactorial, which include tooth morphology, inter-proximal spaces, the distance of inter-proximal contact to the alveolar bone crest, gingival phenotype, patient’s age, and history of periodontitis [12]. Moreover, in terms of OT, a relatively high incidence of GBT (38–43.7%) has been reported following OT. Indeed, this is not commensurate with the present-day high esthetic demands of young patients undergoing OT [9].

The evidence of the association between the incidence of GBT and OT is contradictory [13]. Some studies indicate that OT leads to the development of GBT [14,15,16,17,18,19,20,21,22], while others reported that OT might stimulate interdental papilla formation and accordingly lead to the reduction of GBT incidence [23,24,25]. To the best of our knowledge, the published studies and available evidence scarcely answer the question of whether there is an association between OT and increasing incidence of GBT. Thus, the present systematic review aims to explore this association. Additionally, alveolar bone loss and the risk factors associated with the incidence of GBT following OT have been examined.

## 2. Materials and Methods

### 2.1. Guidelines

The protocol for this systematic review was developed according to the updated Preferred Reporting Items for Systematic Reviews and Meta-Analyses (PRISMA) statement [26]. The study was registered in PROSPERO, an international database of prospectively registered systematic reviews (registration number: PROSPERO 2022 CRD42022315066).

### 2.2. PEO Questions

The research question was developed based on the Population, Exposure, Outcomes (PEO) framework. The focus question was “In individuals ≥ 12 years old, is the treatment with fixed orthodontic appliances associated with increased incidence of GBT after completing the treatment?”:

- Population: Individual ≥ 12 years old.
- Exposure: Fixed orthodontic appliances.
- Outcomes.

Primary outcome: Incidence of GBT.

Secondary outcomes: (1) Alveolar bone loss after OT and its association with GBT and (2) the GBT-associated risk factors.

### 2.3. Search Strategy and Eligibility Criteria

Three electronic databases (Cochrane Central Register of Controlled Trials, Medline via PubMed, and EMBASE via Ovid) were searched for relevant articles published up to March 2022. Combinations of MeSH search terms and text words were used: (“fixed orthodontic treatment *” OR “fixed orthodontic appliance *” OR “orthodontic treatment *” OR “fixed brace *” OR “fixed appliance therap *” OR “fixed brace * treatment *” OR “fixed brace * therap *”) AND (“open gingival embrasure *” OR “gingival embrasure *” OR “gingival black triangle *” OR “black triangle * teeth” OR “ black triangle *” OR “angularis nigra” OR “loss of interdental papilla *” OR “black space” OR “ embrasure” OR “ gingiva” OR “ gingival papilla absence” OR “ interdental black triangle” OR “ interproximal dental papilla”). Additionally, a manual search of bibliographies of the previously published systematic reviews and selected studies were checked for cross-references.

The eligibility criteria included patients exclusively having fixed orthodontic treatment and free from GBT and periodontal diseases at the start of treatment with an age of ≥12 years old. All quantitative studies in the English language were included. Reviews, commentaries, case reports, case series, policy documents, and opinion articles were excluded.

### 2.4. Study Selection and Data Extraction

Based on eligibility criteria, retrieved articles went through a two-step screening procedure. This included titles, abstracts, and full-text screening, according to eligibility criteria, by two independent reviewers (RJZ and GSS). When there is missing or incomplete information, the publications were excluded. Differences between reviewers were addressed by a discussion with the third reviewer (AAA). Inter-reviewer agreement was measured by Cohen’s Kappa test [27].

### 2.5. Data Screening and Extraction

Data including authors’ names and year of publication, the aim and design of the study, the age of participants, the method of assessing the GBT, and the incidence of GBT were retrieved. Additionally, changes in the alveolar bone level following OT and the reported risk factors associated with the incidence of GBT were also recorded.

### 2.6. Outcome Measures

The primary outcome measure is the incidence of GBT, whereas the secondary outcomes are alveolar bone loss after OT and its association with GBT and GBT-associated risk factors.

### 2.7. Quality Assessment

A methodological quality critical appraisal checklist proposed by the Joanna Briggs Institute (JBI) systematic review methods manual [28] was used to assess the risk of bias in individual studies. This tool is dedicated to observational studies reporting prevalence data considering sample frame/recruitment appropriateness, sample size, subject and setting descriptions, data analysis coverage, ascertainment, measurement of the condition, reporting statistical analysis thoroughness, and response rate adequacy and management. Each domain was rated as having a high, low, or uncertain risk of bias. The studies were evaluated separately by two reviewers (MSS and MSZ). Disagreements were discussed and resolved to reach a consensus between the reviewers. The appraisal results were utilized to guide the synthesis and interpretation of the review findings.

The risk of bias (RoB) for each study was categorized, according to the final JBI scores, as follows: ‘high’ for scores ≤ 49%, ‘moderate’ for scores between 50% and 69%, and ‘low’ for scores > 70% [29,30,31].

## 3. Results

### 3.1. Selection of Studies

**Figure 1:**
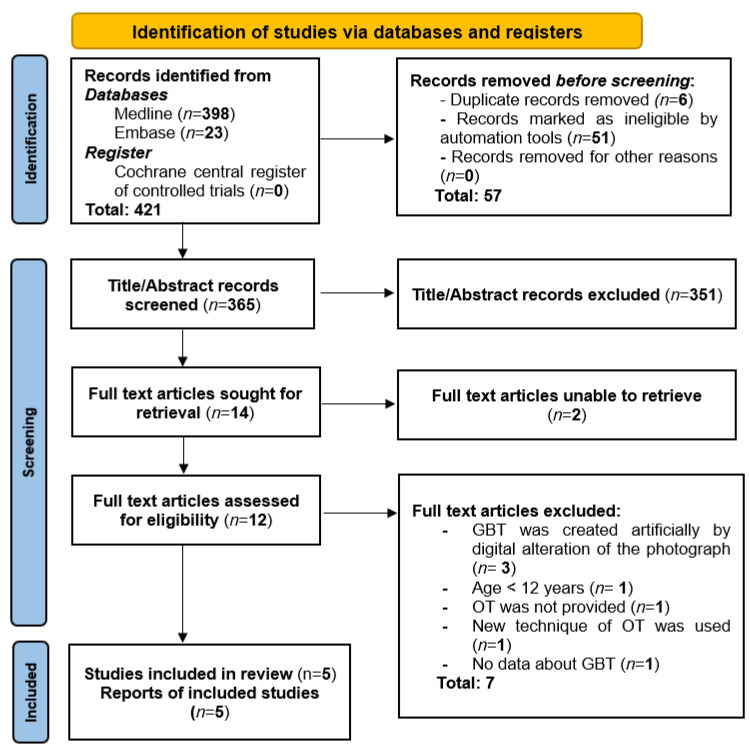
PRISMA flow diagram.

A total of 421 records were found during the search process. After removing duplicates, 365 records remained. Titles and abstracts were screened by two reviewers, which resulted in excluding 351 records. At this step, 14 articles were nominated (Figure 1) for full-text reading, which led to the exclusion of the other nine articles [32,33,34,35,36,37,38,39,40], and the reasons for excluding these records are summarized in Table 1. Finally, five articles [1,10,13,41,42] fulfilling the eligibility criteria were further analyzed for data extraction and answering the PEO question. The computed Cohen’s kappa values for inter-reviewer agreement for the title/abstract and full-text screening procedure were 0.83 and 0.89, respectively.

### 3.2. Study Design and Populations

Amongst the included studies for the final analysis, three were retrospective cohort studies [10,41,42], one was a prospective clinical study [13], and one was a cross-sectional study [1]. The minimum number of patients included was 80 [13] while the maximum was 337 [42]. Regarding the age, the widest range was 20–77 years old [42], the minimum reported mean age was 15 ± 3 years old [41], and the maximum reported mean age was 31.9 years old [42]. The age range was not reported in two studies [10,41], and the mean age was not reported in one study [13].

### 3.3. Study Outcomes

#### 3.3.1. Incidence of Gingival Black Triangle (Primary Outcome)

The incidence of GBT was measured by a diagnostic cast and intraoral photographs in two studies [13,41], a digital image in two other studies [10,42], and clinically using a periodontal probe in another study [1]. Overall, the incidence of GBT in the included studies tends to be high. The lowest incidence of GBT following OT was 38% [42], while the highest incidence was 58% [10]. Two studies reported a GBT incidence of 43% [1,13], and another study reported an incidence of approximately 42% [41] (Table 2).

#### 3.3.2. Alveolar Bone Loss (Secondary Outcome)

The measurement of alveolar bone loss varied in the included studies. Abdelhafez et al. measured bone loss from only the cemento-enamel junction to the alveolar crest [1]. Measuring the distance from the inter-proximal contact (IPC) point to the alveolar crest to estimate bone loss was performed by An et al. [10]. Kurth and Kokich used both methods to measure bone loss [42]. One study measured alveolar bone loss by the distance from the Frankfort plane to the mandibular alveolar crest [13]. Only one study did not report alveolar bone loss [41] (Table 3).

Three studies reported that the reduction of alveolar bone loss was statistically significant following OT and the changes in alveolar bone level associated with the incidence of GBT [1,10,13], whereas one study indicated that alveolar bone loss was not associated with GBT; however, it was found that an increased distance from the alveolar bone to the IPC is correlated with GBT after OT [42] (Table 3).

### 3.4. Risk Factors for Incidence of GBT in Patients Undergoing OT

Data from the included articles lack a consensus on the risk factor(s) associated with the incidence of GBT. In fact, the results were controversial; Ko-Kimura et al. [13] indicated age as a risk factor; however, it was not associated with GBT according to An et al. [10]. One study [42] suggested that the level of alveolar bone is a risk factor for the incidence of GBT. Two studies agreed that the duration of treatment was not associated with the incidence of GBT [10,13]. Likewise, the severity and degree of malocclusion were not reported as risk factors for GBT by two studies [13,42]. Further, two studies [10,42] indicated that tooth-related morphology/dimension was associated with the incidence of GBT. Other reported risk factors varied from the direction of orthodontic movements [10], the size of the embrasure area [42], the width of keratinized gingiva, and the number of missing teeth [1]. Only one study [41] did not report any risk factor(s) in association with GBT (Table 3).

### 3.5. Quality Assessment

The risk of bias for three studies [10,13,41] was assessed as moderate, while for the remaining two studies [1,42], the risk of bias was judged to be low. The response rate criterion was deemed inapplicable for retrospective research where non-response and dropout were unlikely. Similarly, the assessment of this study design was deemed unclear as it was not expressly stated that all patients meeting the inclusion criteria were chosen (Table 4).

## 4. Discussion

The current systematic review aimed to answer the research question of “whether OT with fixed appliances influences the incidence of GBT” by synthesizing and analyzing evidence from available empirical studies. Indeed, the interproximal papilla plays a key role in protecting the periodontal structures from microbial invasions in addition to being an integral part of phonetics and esthetics [43]. Consequently, the loss of interdental papilla leaves a triangular space between the teeth, allowing the passage of air or saliva, causing an embarrassing esthetic problem, food impact, and periodontal problems [44]. It is important to take into consideration the fact reconstruction of the lost interdental papilla is one of the least predictable and most challenging procedures. For example, coronally repositioning the flap and connective tissue flap are still considered the gold standard in regenerating the lost periodontal tissues; however, these procedures are usually associated with disadvantages such as increased donor site morbidity, prolonged surgical times, and increased chances of patient withdrawal [11,25]. Thus, it is very important to avoid/minimize any trauma during dental procedures to maintain papillary integrity [43]. Available literature indicates that GBT increases by approximately 58% following OT [10]. Undoubtfully, outcomes of successful OT are typically determined by the esthetic outcome [45].

The primary outcome of this review was to determine the incidence of GBT following OT. Extracted data from the studies included in the final analysis showed that GBT incidence ranged from 38% [42] to 58% [10]. This variation could be attributed to differences in study designs, sample sizes, age range, and methods used for assessing GBT. In brief, the design followed by three studies was a retrospective cohort [10,41,42], one study was a prospective clinical study [13], and one was a cross-sectional study [1]. The sample size ranged from 80 [13] to 337 patients [42], while the mean age ranged from 15 ± 3 [41] to 31.9 years [42]. Vast variations in the aforementioned variables could dramatically alter the incidence of GBT.

Other findings (secondary) in this review included the level of alveolar bone following OT and its association with the incidence of GBT. Three studies showed that the reduction of the alveolar bone level was statistically significant following OT, and these changes were associated with the incidence of GBT [1,10,13]. Bone loss and the reduction of the alveolar bone support in general and the reduction of interproximal bone height are common problems associated with OT [46,47,48]. It is generally accepted that a distance over 5 mm between the IPC point and the crest of the alveolar bone is associated with GBT after OT [49]. An et al. [10] observed that GBT was formed when increasing the distance between the IPC point and alveolar bone crest when the latter undergoes resorption. This was consistent with the results of Kurth and Kokich [42] who suggested that a short and more incisally positioned IPC point together with a divergent or triangular-shaped crown are risk factors for developing GBT. However, the same study stated that an increased distance from the alveolar bone to the IPC after OT is correlated to GBT and not alveolar bone loss [42]. Ko-Kimura et al. [13] considered that age greater than 20 years old is a factor in increasing the incidence of GBT, which is logically explained by the slower healing capacity affecting proliferative, inflammatory, and bone processes with increasing age [50]. Lastly, Abdelhafez et al. [1] found an association between the change in width of keratinized gingiva and the number of extracted teeth and the risk of GBT development. The thick periodontal biotype exhibits high vascularity and biologic capacity to heal when subjected to external stimuli. Conversely, these features in the thin periodontal biotype are inferior to the thicker biotype, therefore more prone to recession and loss of attachment [51].

As with any other health issue, multiple risk factors could be associated with GBT and impose a detrimental effect on the development and progression of this condition. The study of Kurth and Kokich [42] showed a strong association between divergent roots and GBT and stated that a 1° increase in root divergence is directly proportional to a 14% to 21% increase in GBT. In addition, the development of GBT is affected by the labiolingual thickness of the supporting bone and soft tissue. For instance, labial movement, i.e., the proclination of teeth, leads to thinning of the supporting bones and gingival tissues on the labial surface of the teeth and migration of these tissues, apically causing GBT formation [36]. Results from one of the included studies [10] supported this notion, in which horizontal movement in the maxilla was considered a risk factor for the development of GBT. This could be explained by the fact that the facial bone plate of the maxilla is relatively thin accompanied by prominent roots of maxillary teeth. For instance, the thickness of the labial plate at maxillary central incisors, lateral incisors, and canines is 0.97 ± 0.18 mm, 0.78 ± 0.21 mm, and 0.95 ± 0.35 mm, respectively, while the thickness of the labial plate over their mandibular counterparts is 0.86 ± 0.59 mm, 0.88 ± 0.70 mm, and 1.17 ± 0.70 mm, respectively [52].

Additionally, the severity of crowding contributes to increasing the incidence of GBT. Ko-Kimura et al. [13] found that GBT equally developed in patients with crowding of less than 4 mm and 4 to 8 mm. However, the odds ratio was increased by 7% in patients exhibiting crowding of more than 8 mm. This means an increase of 1 mm of crowding beyond 8 mm will increase the chances of GBT by 7%, i.e., a patient with 12 mm crowding has an odds ratio of 28% of developing GBT following OT. Moreover, a large diastema closure during OT is mostly associated with GBT formation because the volume of soft tissues in the gingival embrasure after OT depends on the available tissue and bone levels [11]. Notably, patients with triangular crown morphology at the start of OT were more susceptible to GBT when the treatment was completed [11]. This particular crown morphology is characterized by a narrow cervical and wide incisal area resulting in an aberrant contact point located more incisally. Therefore, special attention is required to convert this contact point into the contact area to avoid the formation of an open gingival embrasure [11].

The main limitation of the current study is that most of the included studies were observational studies. Although this type of study has a low level in the hierarchy of evidence, they are considered pragmatic studies that can show the real-life impact and are more likely to show significant clinical problems. Additionally, due to the heterogeneity of the methods in the included studies, it was not possible to standardize the severity of malocclusion at baseline, the type of fixed appliance used, the duration of the treatment, and the age range. Nevertheless, OT with a fixed appliance could be associated with GBT; therefore, it is recommended that the clinician should consider this problem during OT planning and discuss it with the patient at the start of treatment with potential risk factors being taken into consideration.

## 5. Conclusions

Although analyses from this systematic review suggested an increased incidence of GBT after OT, no concrete conclusion can be withdrawn due to the heterogeneity of data. Similarly, no conclusion could be outlined regarding the risk factors associated with GBT due to the variations and discrepancies in the reported results across the included studies. Therefore, to exclude the effect of risk factors, further high-level randomized control trials are essential to reach a solid conclusion on the effect of OT on the development of GBT.

## Figures and Tables

**Table 1:** Reasons for exclusion after full-text reading.

No.	Author, Year	Reason(s) for Exclusion
**1**	Uribe et al., 2011 [32]	GBT was artificially created by the digital alteration of the photograph
**2**	Pithon et al., 2012 [33]	
**3**	Bolas-Colvee et al., 2018 [34]	
**4**	Ikeda et al., 2004 [35]	Full text not available
**5**	Kandasamy et al., 2007 [36]	
**6**	McMorrow and Millett, 2017 [37]	No orthodontic treatment provided
**7**	Jeong et al., 2016 [38]	Age < 12
**8**	Vilhjálmsson et al., 2019 [39]	New technique in OT provided
**9**	Jamilian et al., 2015 [40]	GBT was only indicated as present or absent

GBT: Gingival black triangle, OT: Orthodontic treatment.

**Table 2:** Incidence of gingival black triangle following orthodontic treatment (primary outcome).

Author, Year	Aim	Study Design/Sample	Age (Years)	Assessment	Incidence of GBT
Burke et al., 1994 [41]	To determine: (1) The incidence of overlapped and crowded MCI; (2) the incidence of OGE spaces (GBT) after orthodontic alignment of crowded MCI, and (3) the width of the gingival base of that triangular space.	Retrospective cohort Patients with crowded MCI (*n* = 129)	Mean: 15 ± 3 Range: NR	Diagnostic casts and photographs. The dimension of the embrasure was measured horizontally at the most cervical aspect of the triangular space (within 0.017 mm).	41.9% (*n* = 54)
Kurth and Kokich, 2001 [42]	(1) To determine the prevalence of posttreatment OGE in adult orthodontic patients. (2) To examine the association of pre-treatment maxillary incisor malalignment, posttreatment alveolar bone height, interproximal contact position, root angulation, crown shape, and embrasure area with OGE.	Retrospective cohort Adult orthodontic patients (*n* = 337)	Mean: 31.9 Range: 20 to 77	Occlusal digital images of the pre-treatment maxillary models analyzed by software for MCI overlap and rotation. Posttreatment, digital image of radiographs to assess alveolar bone height, interproximal contact height, crown shape, root angulation, the long axis of the tooth, and embrasure area.	38% (*n* = 128)
Ko-Kimura et al., 2003 [13]	To determine: (1) the prevalence of OGE in a group of orthodontic patients; (2) if OGE is related to pre-treatment age, the severity of mandibular incisor crowding, duration of treatment, and/or changes in alveolar bone height.	Prospective clinical trial Patients with Class I malocclusion (*n* = 80) Distribution according to: - Sex; male (*n* = 33), female (*n* = 47) - Age groups; 15 to 20 years (*n* = 38), >20 years (*n* = 42)	Mean: NR Range: 15 to 31	Study casts and intra-oral photographs Heights and widths of the five gingival embrasures, from the mesial surface of one mandibular canine to the mesial surface of the contralateral canine, were measured with dial calipers to the nearest 0.01 mm. GBT definition: width ≥ 1.0 mm, height ≥ 2.0 mm	43.7% (*n* = 35)
An et al., 2018 [10]	To determine the incidence of OGE after OT and to examine the predisposing factors in combination with OT.	Retrospective cohort *n* = 100 (Male = 29, female = 71)	Mean: 24.7 ± 67.6 Range: NR	Frontal intraoral photographs, lateral cephalograms, and periapical radiographs were taken with a 4 mm metal bead, and study models. OGE is subdivided into mild, moderate, and severe groups.	Overall: 58% (*n* = 58) Mild: 45% (*n* = 45) Moderate: 13% (*n* = 13) Severe: 0% (*n* = 0)
Abdelhafez et al., 2022 [1]	To assess possible positive/negative effects of OT on the periodontium and tissue esthetics.	Cross-sectional study Patients completed OT (*n* = 156) - With extraction of teeth (*n* = 51, 32.6%) - Without extraction of teeth (*n* = 105, 67.3%)	Mean: 21.47 ± 3.5 Range: 18 to 39	The height of papilla, the width of keratinized gingiva, gingival recession, degree of tooth display, smile line, crestal bone level, and proximal caries were assessed using a Michigan O periodontal probe with William’s grading	Overall: 43% (*n* = 68) Extraction group: 45.1% (*n* = 23) Non-extraction: 42.1% (*n* = 45)

MCI: Maxillary central incisors, NR: Not reported, GBT: Gingival black triangle, OGE: Open gingival embrasures, OT: Orthodontic treatment.

**Table 3:** Alveolar bone level (secondary outcomes) in subjects undergoing orthodontic treatment.

Author, Year	Inclusion Criteria	Detail of Measurement	Baseline Bone Level (mm)	Post Treatment Bone Level (mm)	∆ Mean Bone Loss Difference (mm)	Conclusion	Reported Risk Factor(s) in Association with GBT
Burke et al., 1994 [41]	Presence of six maxillary anterior teeth.	NR	NR	NR	NR	NR	NR
Kurth and Kokich, 2001 [42]	At least 20 years old at the start of orthodontic treatment, there were no restorations or alterations of the mesial surfaces of the maxillary central incisors. Post-treatment frontal intraoral photographs were available.	Bone height measured from CEJ to alveolar crest/ alveolar bone to IPC point.	2.28 ± 1.93/ 5.50 ± 2.32	1.95 ± 1.74/ 7.01 ± 2.24	−0.33 ± 0.72/ −1.51 ± 0.93	Alveolar bone loss not associated with GBT/an increased distance from the alveolar bone to the IPC is correlated with GBT after orthodontic therapy.	Associated: Root angulation Alveolar bone to IPC distance. Divergent or triangular-shaped crown. Increased embrasure area. Not associated: Pre-treatment maxillary central incisor rotation and overlap.
Ko-Kimura et al., 2003 [13]	Class I malocclusion with severity of crowding of <4 mm, 4–8 mm and >8 mm.	Bone loss measured by distance from the Frankfort plane to the mandibular alveolar crest.	65.3 ± 5.72	68.7 ± 5.5	−3.43 ± 0.15	GBT were associated with resorption of the alveolar crest following orthodontic treatment.	Associated: Age > 20 years old. Not associated: Duration of treatment (<3 years, >3 years). Severity of crowding (<4 mm, 4–8 mm, >8 mm). Distance of Frankfort plane to the incisal edge of the most prominent mandibular incisor.
An et al., 2018 [10]	Presence of central incisors and all types of malocclusion.	The distance between the mesial CEJs of two central incisors was measured from IPC point to the alveolar bone crest.	Maxilla: 5.04 ± 0.91 Mandible: 4.90 ± 0.87	Maxilla: 5.51 ± 0.95 Mandible: 5.85 ± 1.05	Maxilla: −0.47 ± 0.84 Mandible: −0.95 ± 1.03	A large distance from the IPC point to the alveolar crest after treatment can cause GBT after orthodontic treatment.	Associated: Crown ratio in the mandible. Vertical movement in the mandible. Horizontal movement in the maxilla. Not associated: Age. Crowding degree. Treatment duration. Crown ratio in the maxilla. Vertical movement in the maxilla. Horizontal movement in mandible.
Abdelhafez et al., 2022 [1]	Mild malocclusion, and extraction may or may not be part of orthodontic treatment and orthodontic treatment at least 6 months ago.	The crestal bone level was measured on radiographs at the mesial and distal surfaces of all teeth as the distance from the CEJ to the crest of the alveolar bone.	NR	NR	Upper anterior teeth: Ortho treated 1.91 ± 0.39 vs. non-ortho treated 1.78 ± 0.35 Lower anterior teeth: ortho treated 2.15 ± 0.54 vs. non-ortho treated 1.98 ± 0.51	The orthodontic treatment appeared to be associated with crestal bone levels in relation to the CEJ at upper and lower anterior teeth.	Associated: Width of keratinized gingiva. Number of extracted teeth. Not associated: Proximal caries. Gingival recession. Smile line.

NR: Not reported, GBT: Gingival black triangle, IPC: Interproximal contact, CEJ: Cemento-enamel junction.

**Table 4:** The critical appraisal results of the included studies using the JBI-prevalence critical appraisal checklist.

Study	Was the Sample Frame Appropriate to Address the Target Population?	Were Study Participants Sampled in an Appropriate Way?	Was the Sample Size Adequate?	Were the Study Subjects and the Setting Described in Detail?	Was the Data Analysis Conducted with Sufficient Coverage of the Identified Sample?	Were Valid Methods Used for the Identification of the Condition?	Was the Condition Measured in a Standard, Reliable Way for All Participants?	Was There Appropriate Statistical Analysis?	Was the Response Rate Adequate, and If Not, Was the Low Response Rate Managed Appropriately?	Total % of Yes	Overall Risk of Bias
Burke et al., 1994 [41]	Yes	Unclear	NA	Yes	NA	Yes	Yes	Unclear	NA	66%	Moderate
Kurth and Kokich, 2001 [42]	Yes	Yes	NA	Yes	NA	Yes	Yes	Yes	NA	100%	Low
Ko-Kimura et al., 2003 [13]	Yes	Unclear	Unclear	Yes	Yes	Yes	Unclear	Yes	Yes	66%	Moderate
An et al., 2018 [10]	Yes	Yes	NA	Yes	NA	Yes	Yes	Yes	NA	66%	Moderate
Abdelhafez et al., 2022 [1]	Yes	Yes	Yes	Yes	Yes	Yes	Yes	Yes	Yes	100%	Low

NA: not applicable.

## Data Availability

The data presented in this study are available on request from the corresponding author.
